# A putative HIV-1 subtype C/CRF11_cpx unique recombinant from South Africa

**DOI:** 10.1186/s40064-016-1924-z

**Published:** 2016-03-05

**Authors:** Pascal Obong Bessong, Benson Iweriebor

**Affiliations:** HIV/AIDS and Global Health Research Programme, Room FF172 Life Sciences Building, Department of Microbiology, University of Venda, Thohoyandou, 0950 South Africa; Department of Biochemistry and Microbiology, University of Fort Hare, Alice, South Africa

**Keywords:** HIV-1 subtype C, CRF11_cpx, Recombinant, HIV subtyping, South Africa

## Abstract

The HIV epidemic in South Africa is overwhelmingly driven by HIV-1 subtype C viruses. The HIV *gag*, *pol*, *env* (C2-V5) and *nef* sequences of virus 08MB26ZA, obtained from a 47 year old woman, were studied by phylogenetic analysis, REGA and the jumping Profile Hidden Markov Model (jPHMM) tools. The *pol* gene was further analyzed for recombination by Simplot. The *pol* and *env* sequences were examined for genetic drug resistance mutations and predicted co-receptor usage respectively. There was agreement in the assignment of the *gag* sequence as pure HIV-1 subtype C by phylogenetic, REGA and jPHMM analyses. The *pol* sequence clustered with CRF11_cpx and in the J-clade by phylogenetic analysis; and to a CRF11_cpx/subtype C recombinant by REGA. The assignment of *pol* to CRF11_cpx and subtype C was confirmed by Simplot. The recombinant was of the R5 biotype, with no important drug resistance mutations in the *pol* region. The epidemiologic and biologic significance of the virus are unknown. The finding suggests that complex viruses are being introduced into South Africa with potential implications for diagnosis. This is apparently the first report from South Africa of a putative unique recombinant involving CRF11_cpx and subtype C genomes.

## Background

Genetic variants of HIV contribute to the pandemic worldwide and have potential consequences for diagnostics, treatment and treatment monitoring, and the development of an effective vaccine (Hemelaar 2013). Several factors account for the high genetic diversity of HIV: rapid replication turnover, in vivo host selective immune and treatment pressure, and recombination events during replication (Liitsola et al. 1998; Corbet et al. 2000; Ramirez et al. 2008; Taylor et al. 2008). Due to this variability, HIV is classified into types 1 and 2; with type 1 further classified into groups M, O, N and P. Group M, which is responsible for the global HIV-1 epidemic, comprises nine recognized phylogenetic subtypes (A–D, F–H, J, and K), with further divisions into sub-subtypes for A and F viruses.

The emergence of circulating recombinant forms (CRFs) in the global HIV-1 pandemic is important, as together with unique recombinant forms (URF) represent about 20 % of infections (Hemelaar et al. 2011). A complex (cpx) variant represents a recombinant derived from genes of three pure subtypes. CRF11_cpx involving subtypes A, G, J and CRF01-AE is observed in the Republic of Congo, Cameroon and Central African Republic (Wilbe et al. 2000; Montavon et al. 2002). Analysis of partial sequences suggests the presence of CRF11_cpx in Senegal and Gabon (Paraskevis et al. 2000). Subtype J was discovered in 1995 and originated most probably in the Democratic Republic of Congo. Few subtype J sequences are available, and these are mostly from the Democratic Republic of Congo, Cameroon, and Senegal (Los Alamos Database 2014). However, fragments of subtype J are present in many mosaic recombinant forms originating from West Africa (CRF06_cpx) and Central West Africa (CRF11_cpx, CRF13_cpx, and CRF49_cpx), suggesting that this subtype, either in a pure or in a recombinant form, is probably prevalent across Central and West Africa (Laukkanen et al. 1999). Subtype J sequences have also been reported in Uganda, Angola, Zambia, Cuba, Spain and France (Trask et al. 2002; Yebra et al. 2013; Bartolo et al. 2009; Kiwanuka et al. 2009).

Human immunodeficiency type 1 subtype C is overwhelmingly responsible for the epidemic in South Africa, although over time, other subtypes such as A, D, CRF01_AE, inter-subtype and URF such as A1/C, A1/D have been identified (Bredell et al. 2002; Iweriebor et al. 2011; Wilkinson and Engelbrecht 2009). However, CRF11_cpx has not been reported from South Africa. In this report, a plausible recombinant, comprising sequences from HIV-1 subtype C and CRF11_cpx identified in a South African patient is described.

## Results and discussion

### Phylogenetic analyses of gene regions

In an initial phylogenetic analysis, the partial *pol* sequence [complete protease (PR) and first 900 bp of the reverse transcriptase (RT)] clustered with CRF11_cpx reference sequences. Additional recombination analysis with REGA tool revealed a mosaic pattern of unclassified regions alternating with subtype J regions (Nwobegahay et al. 2011). Consequently, an attempt was made to further elucidate the genetic makeup of the virus.

**Fig. 1:**
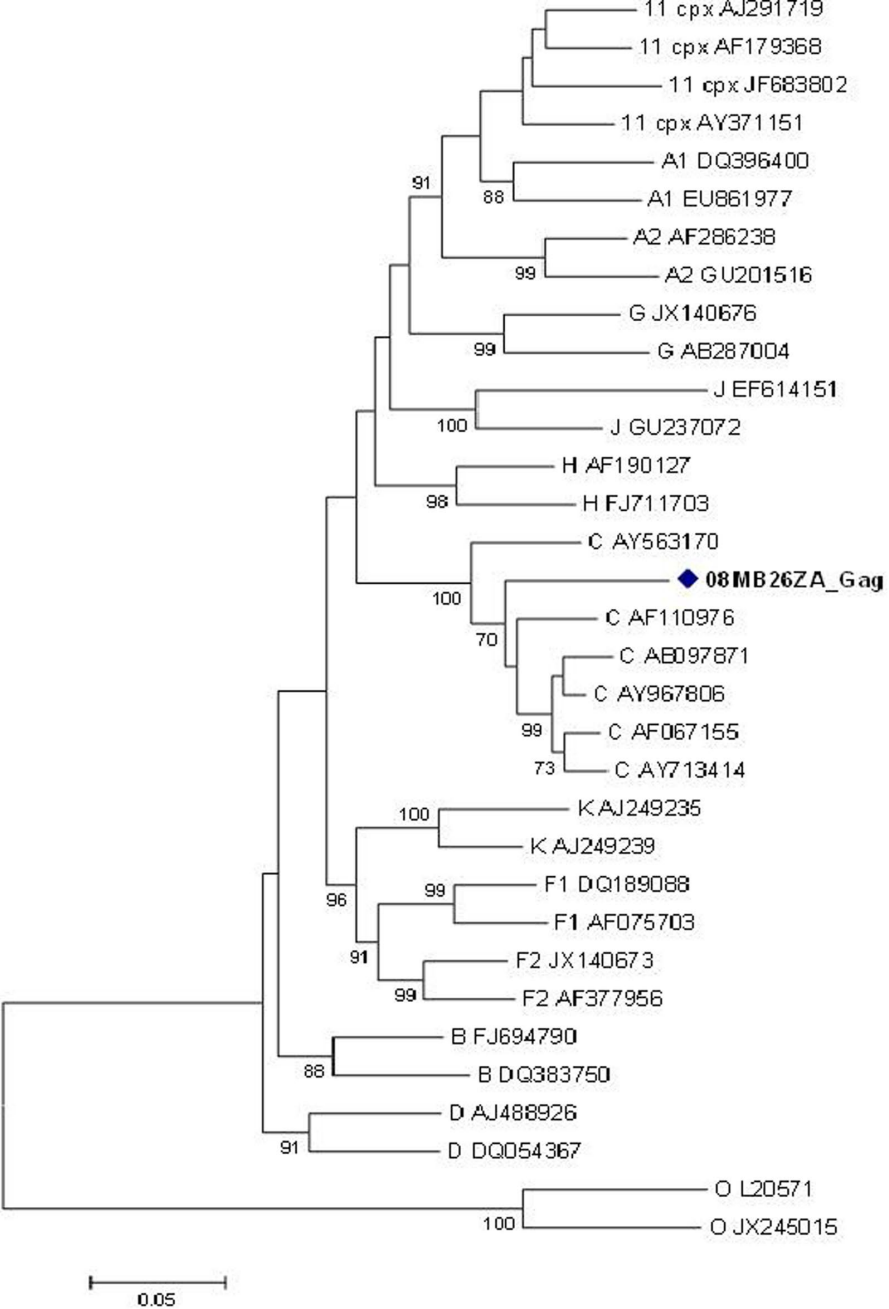
Maximum likelihood phylogenetic analysis of the *gag* sequence of virus 08MB26ZA. The sequence is shown to cluster with HIV-1 subtype C reference sequences. Bootstrap values above 70 % are shown. The tree is rooted with group O sequences. The *scale* represents the number of base substitutions per site

Attempted amplification of the complete genome of sample 08MB26ZA was not successful. Hence gene fragments were generated and directly sequenced and the following reliable sequence lengths were obtained: *gag* sequence (1436 nucleotides, position 789–2252), *pol* (2506 nucleotides, position 2252–4758), e*nv* C2-V5 (719 nucleotides, position 7100–7819), and *nef* (616 nucleotides, position 8792–9413), positions with reference to HXB2 nucleotide coordinates. Phylogenetically, the *gag* sequence clustered with HIV-1 subtype C reference sequences with a bootstrap value of 70 % (Fig. 1).

**Fig. 2:**
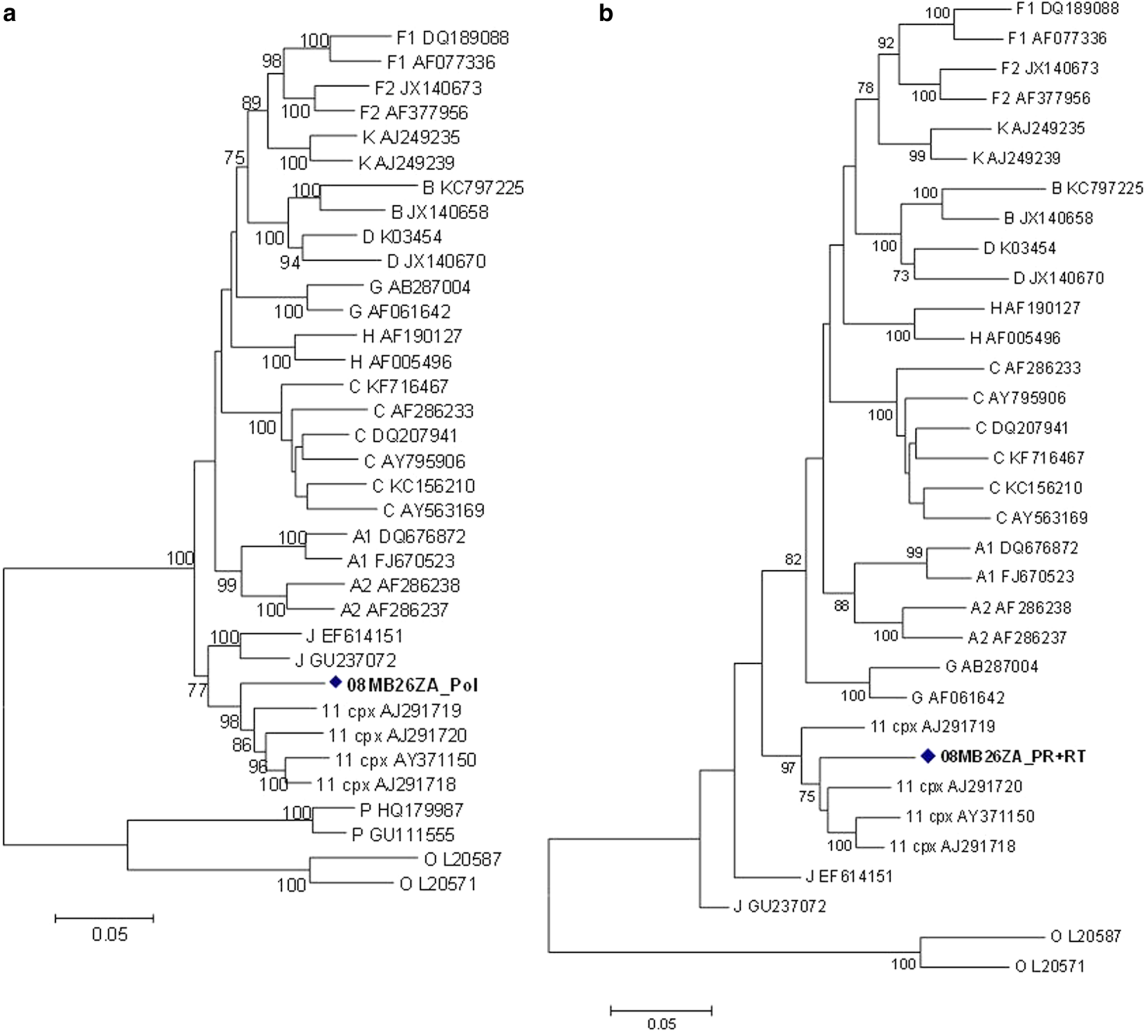
**a** Maximum likelihood phylogenetic tree of the *pol* sequence of virus 08MB26ZA. The sequence is shown to cluster with CRF11_cpx reference sequences with a bootstrap value of 98 %. The tree is rooted with group O sequences. The *scale* represents the number of base substitutions per site. **b** Phylogenetic relationship of combined protease and reverse transcriptase fragments of the polymerase gene of 08MB26ZA. The fragment is assigned to CRF11_cpx with a high bootstrap value. The *scale* represents the number of base substitutions per site

**Fig. 3:**
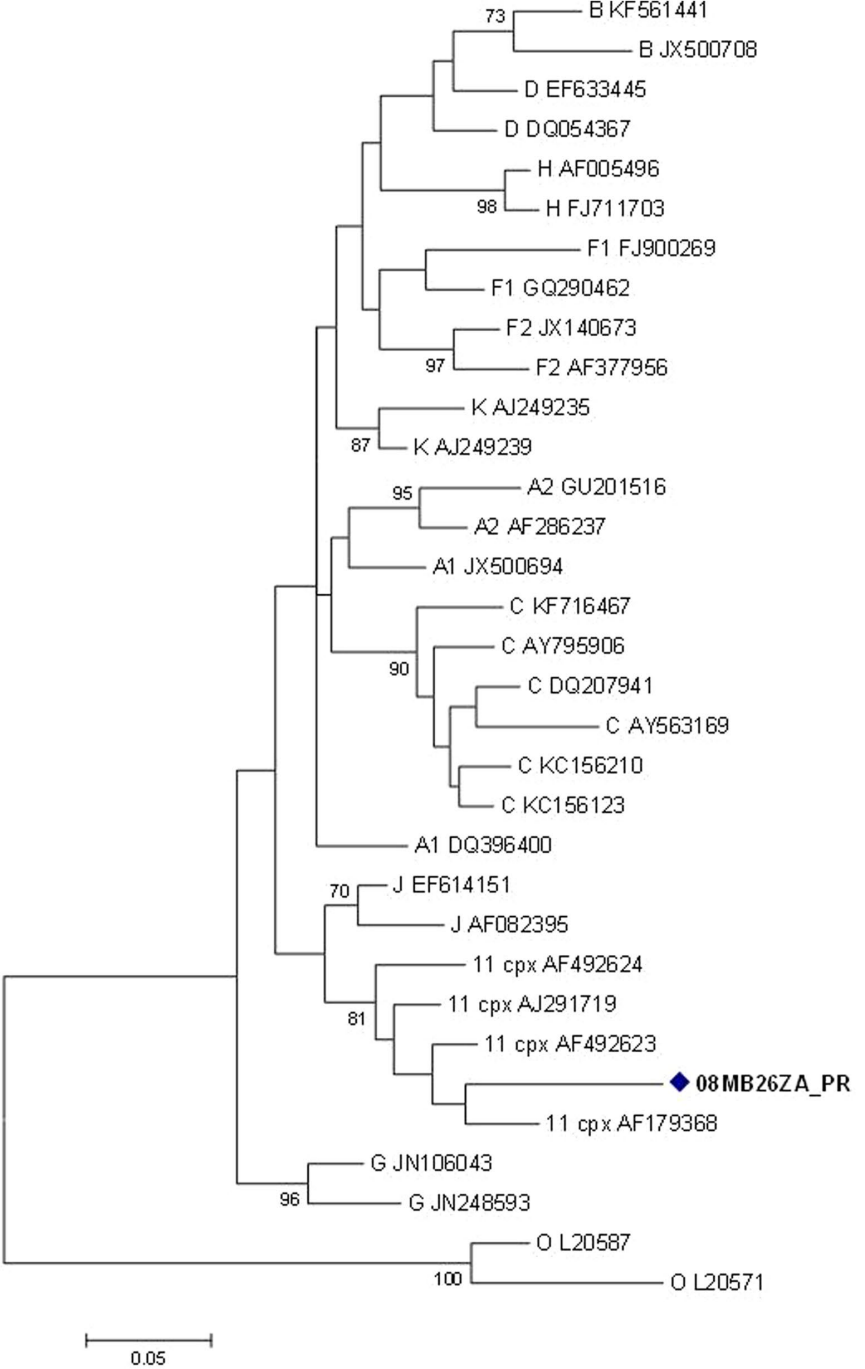
Maximum likelihood phylogenetic analysis of the protease sequence of virus 08MB26ZA. The sequence is shown to cluster with CRF11_cpx reference sequences. Bootstrap values above 70 % are shown. The tree is rooted with group O sequences. The *scale* represents the number of base substitutions per site

**Fig. 4:**
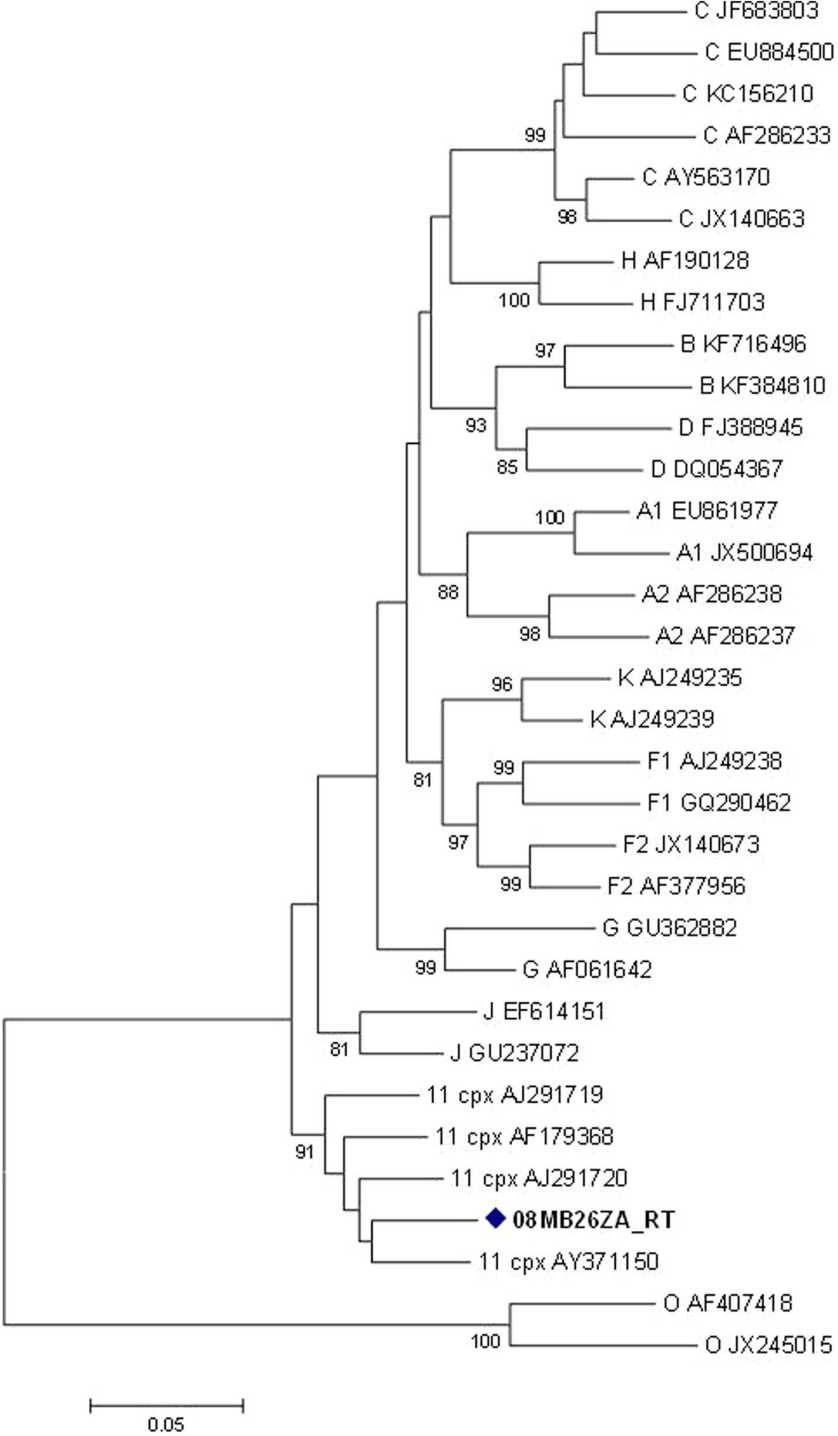
Maximum likelihood phylogenetic analysis of the RT sequence of 08MB26ZA. The sequence clusters with HIV-1 CRF11_cpx reference sequences. Bootstrap values above 70 % are shown. The tree is rooted with group O sequences. The *scale* represents the number of base substitutions per site

**Fig. 5:**
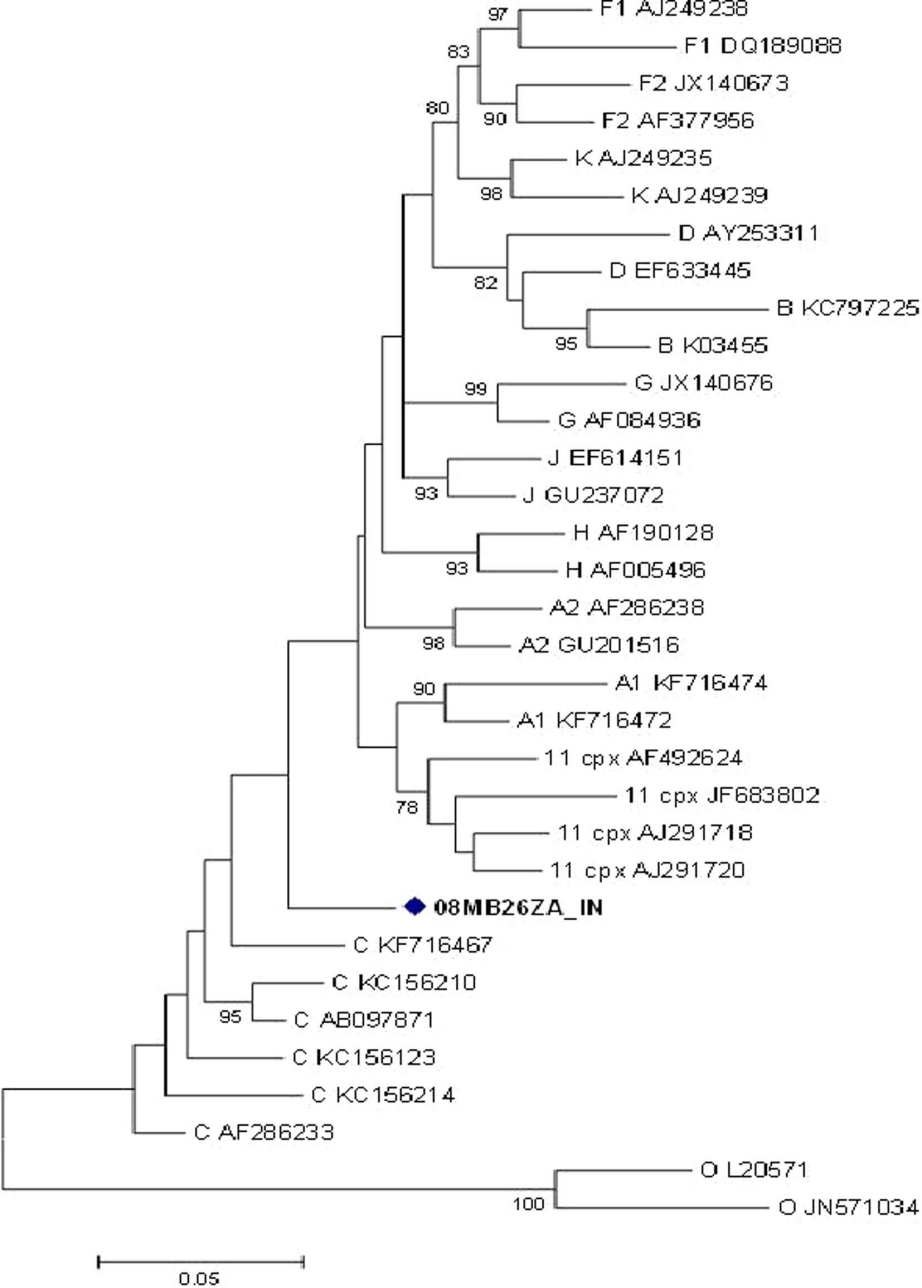
Maximum likelihood phylogenetic analysis of the integrase sequence of 08MB26ZA. The sequence is not delineated as either related to HIV-1 subtype C or CRF11_cpx reference sequences. Bootstrap values above 70 % are shown. The tree is rooted with group O sequences. The *scale* represents the number of base substitutions per site

**Fig. 6:**
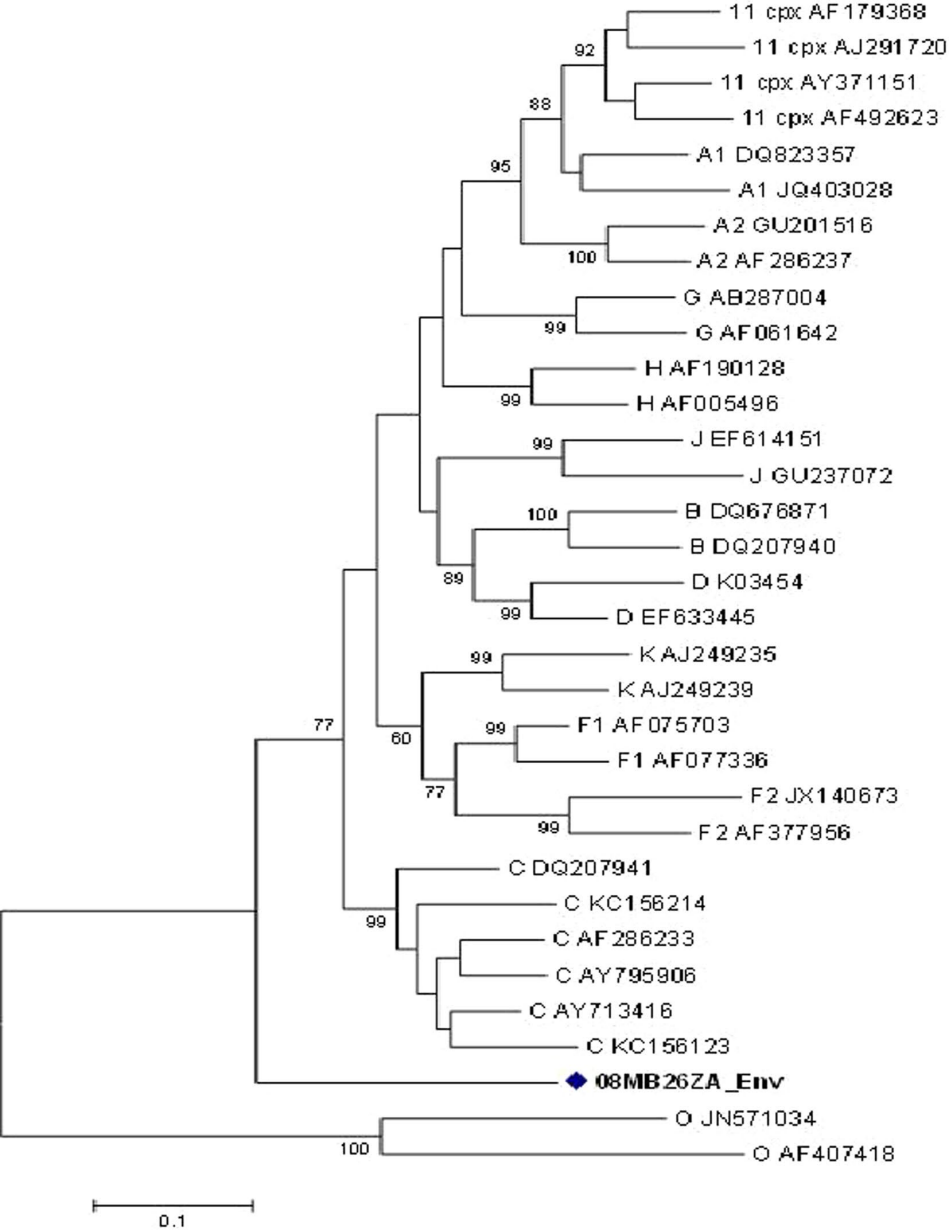
Maximum likelihood phylogenetic analysis of the *env* (C2-V5) sequence of 08MB26ZA. The sequence is shown as untypable. Bootstrap values above 70 % are shown. The tree is rooted with HIV-1 group O sequences. The *scale* represents the number of base substitutions per site

**Fig. 7:**
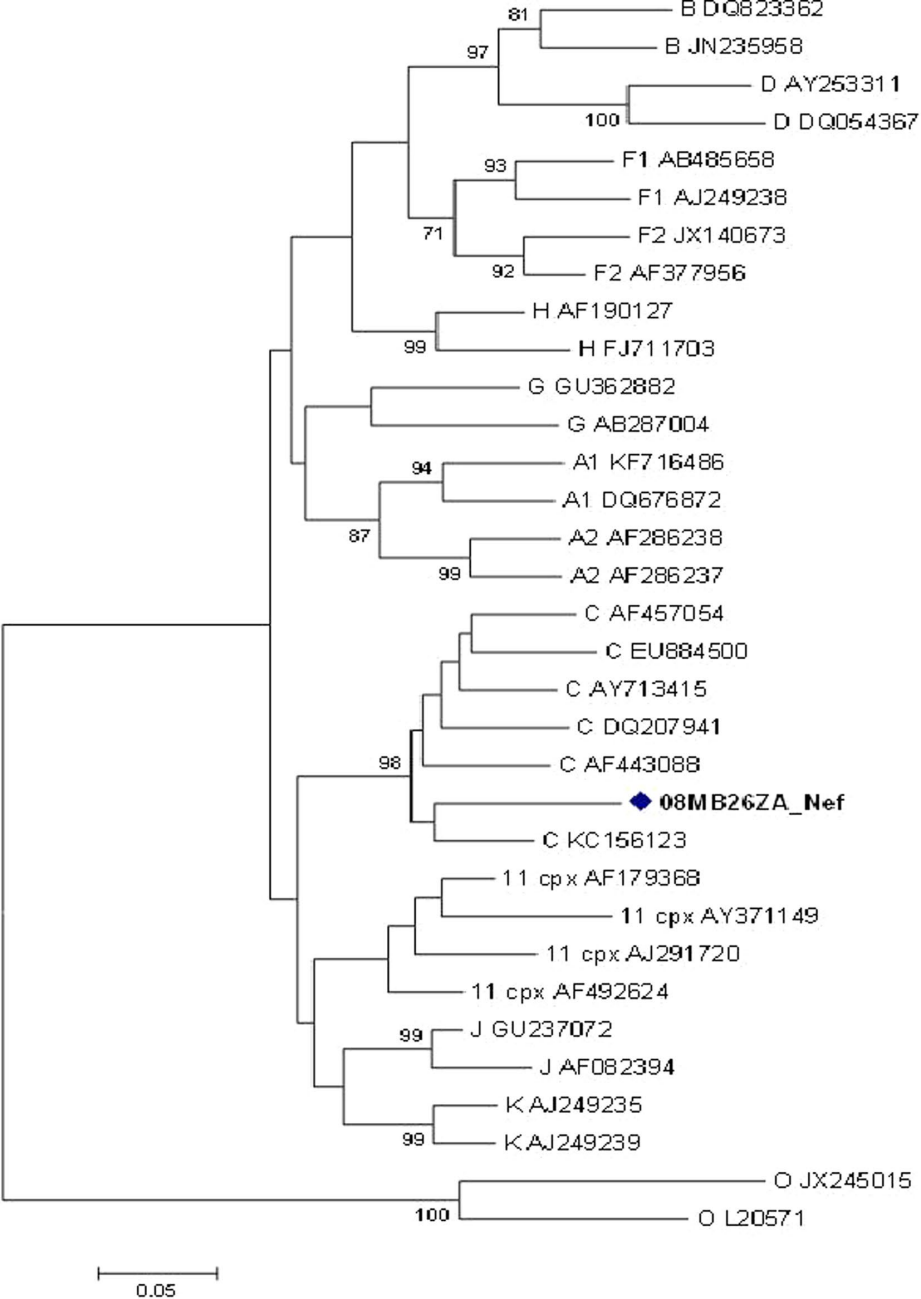
Maximum likelihood phylogenetic analysis of the *nef* sequence of 08MB26ZA. The sequence clusters with HIV-1 subtype C reference sequences. Bootstrap values above 70 % are shown. The tree is rooted with group O sequences. The *scale* represents the number of base substitutions per site

The *pol* gene (i.e. PR + RT + integrase (IN)) clustered with CRF11_cpx sequences with a bootstrap value of 98 %, in the subtype J clade (Fig. 2a). However, without the IN region, the *pol* (i.e. PR + RT) clustered with CRF11_cpx sequences (Fig. 2b). In addition, the PR, RT and IN sequences were individually analyzed: the PR and RT sequences clustered with CRF11_cpx (Figs. 3, 4); and the IN sequence is related to HIV-1 subtype C sequences, but this was not supported by a high bootstrap value (Fig. 5). The *env* (C2-V5) sequence showed a close relation to subtype C sequences with a bootstrap value of 77 %, but not definitively typable (Fig. 6), while the *nef* sequence clustered with subtype C reference sequences (Fig. 7).

### Subtype and recombination analyses of gag and pol genes by REGA

**Fig. 8:**
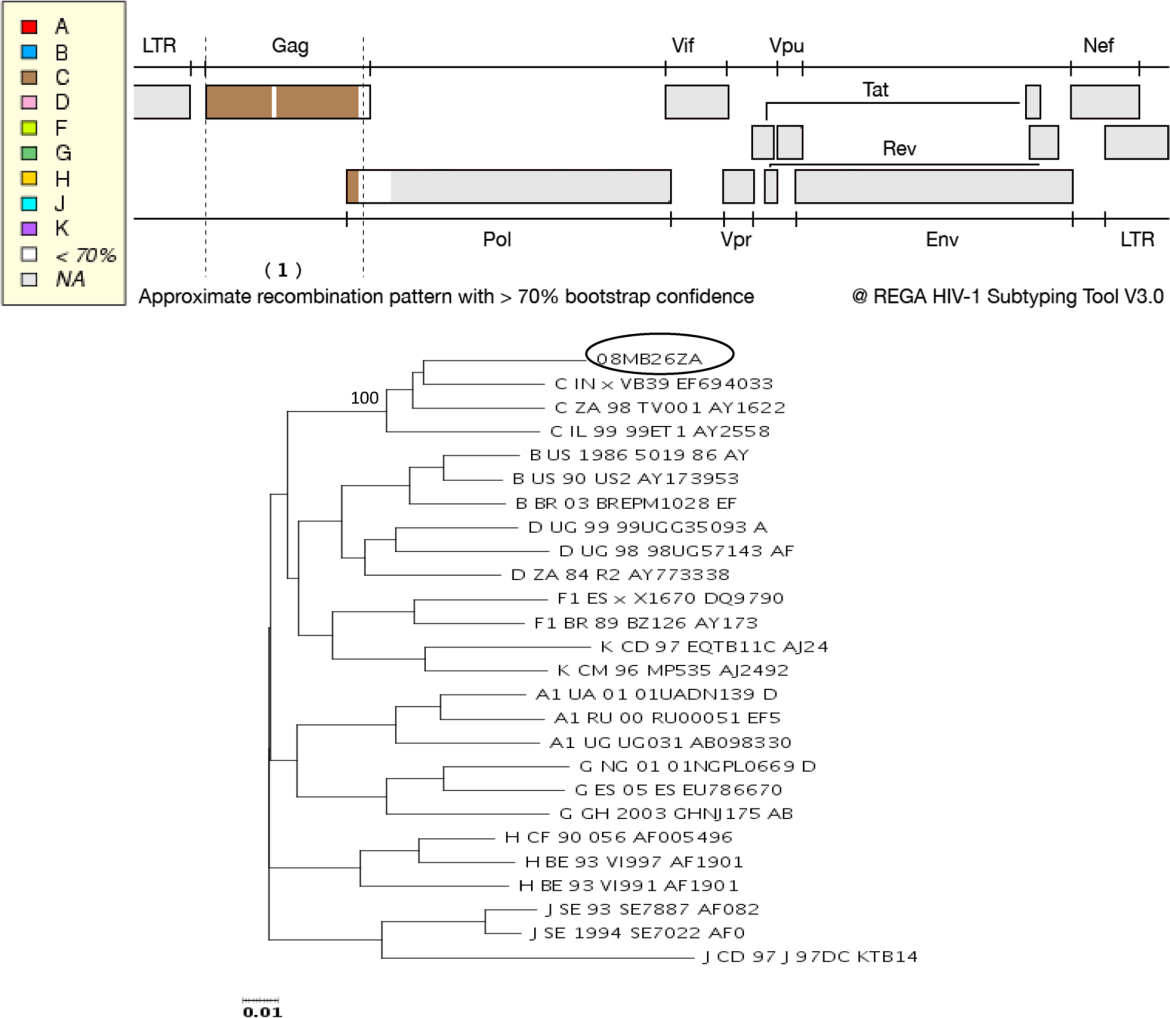
REGA subtype analysis of the *gag* sequence of 08MB26ZA. The sequence is assigned to HIV-1 subtype C with a 100 % bootstrap support

**Fig. 9:**
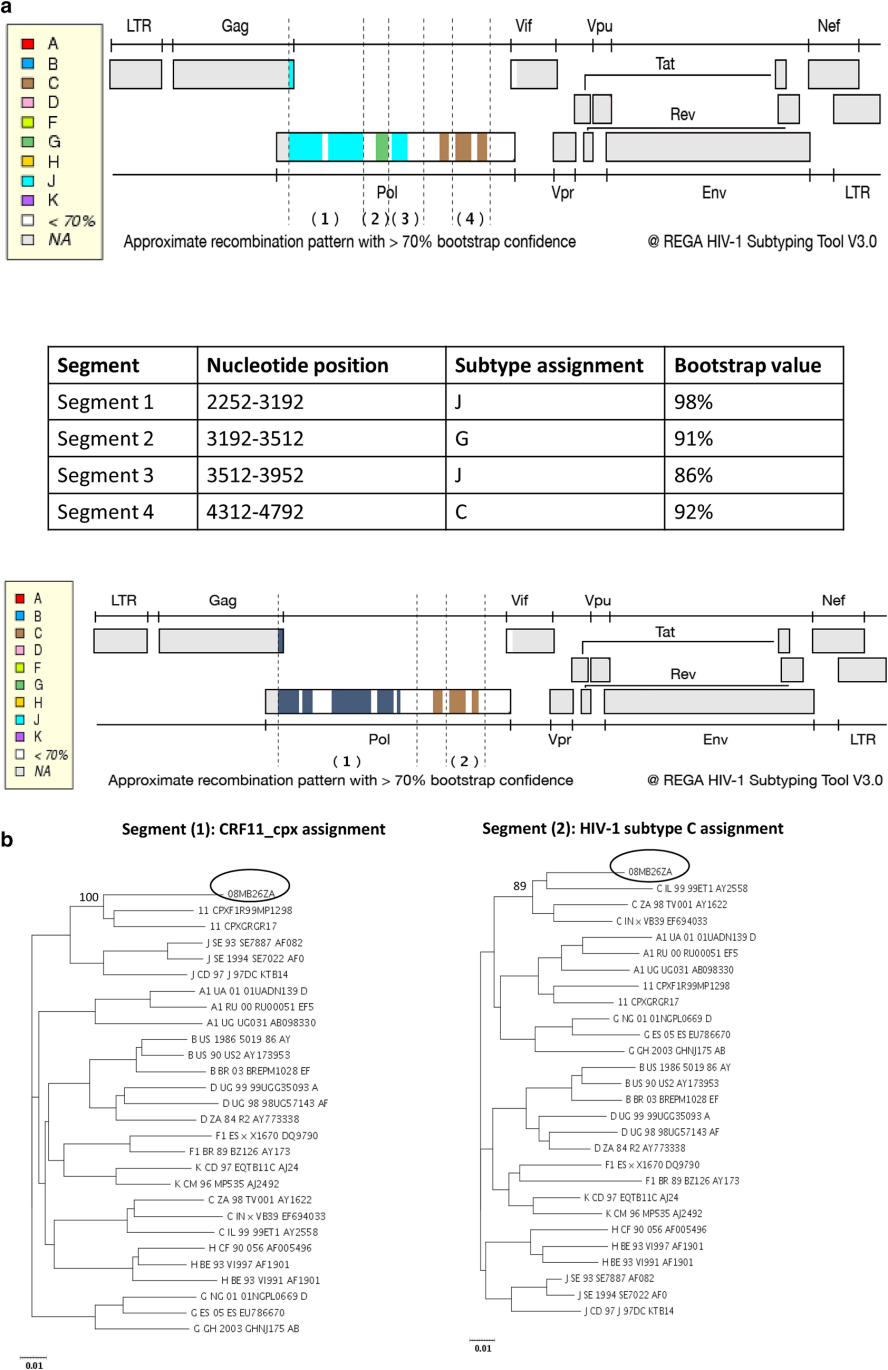
**a** REGA HIV subtype recombination bootscan pattern of the *pol* sequence of 08MB26ZA. The bootscan analysis shows the following patterns: Segment (1) nucleotide position 2252–3192 is assigned to subtype J with 98 % bootstrap confidence; segment (2) nucleotide position 3192–3512 is assigned to subtype G with 91 % bootstrap confidence; section (3) nucleotide position 3512–3952 is assigned to subtype J with 86 % bootstrap confidence; and section (4) nucleotide position 4312–4792 is assigned to subtype C with 92 % bootstrap confidence. **b** REGA HIV-1 subtype/CRF recombination analysis of the *pol* sequence of 08MB26ZA. Segment (1) nucleotide position 2252–3952 is assigned to HIV-1 CRF 11_CPX with a bootstrap support of 100 %; segment (2) nucleotide position 4312–4792 is assigned to HIV-1 subtype C with a bootstrap support of 89 %

Upon HIV subtype analysis with the REGA tool, the *gag* sequence was assigned to HIV-1 subtype C with a bootstrap confidence of >70 % (Fig. 8). On the other hand, the *pol* sequence was shown to contain sequences from subtype J, G, and C in an alternating fashion, with a bootstrap confidence of >70 % (Fig. 9a). Subsequent HIV subtype and CRF recombination analysis with REGA showed that the *pol* was related to both CRF11_cpx (segment 1, Fig. 9b); and HIV-1 subtype C (segment 2, Fig. 9b). The CRF11_cpx relatedness of the *pol* sequence is represented by the PR and RT regions, and the HIV-1 subtype C relatedness is represented by the IN region. This is in agreement with the outcomes of the maximum likelihood phylogenetic analyses of the PR, RT, combined PR and RT, and the IN regions.

The assignment of the *pol* sequence as related to CRF11-cpx/C was according to the REGA schedule 3A which is based on 4 parameters: the query sequence greater than 800 bp, clustering with a CRF with a bootstrap confidence greater than 70 %, coupled with the detection of recombination in the pure subtype bootscan, and further confirmed by CRF bootscan analysis with a confidence of more than 90 % (Peña et al. 2012).

### Subtype and recombination analysis by jPHMM and Simplot

**Fig. 10:**
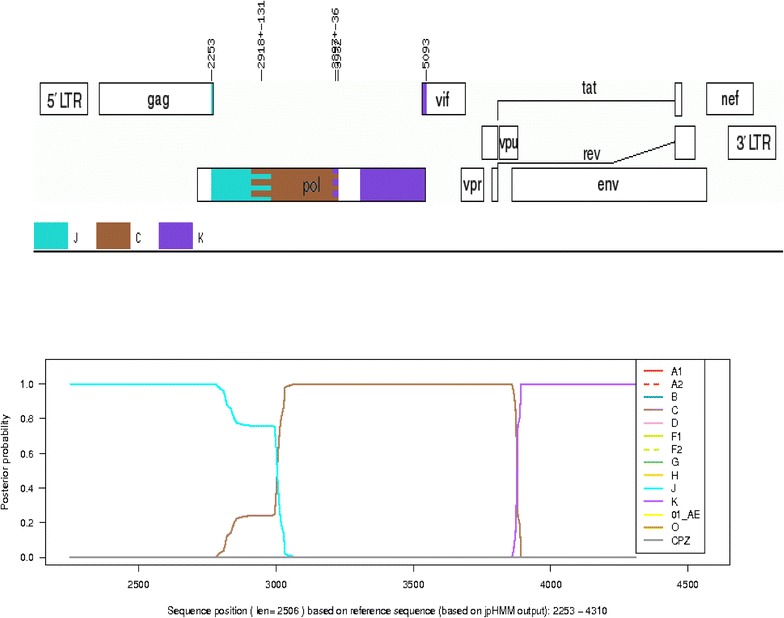
Boostscan representation of the *pol* sequence of 08MB26ZA by jPHMM analysis. The presence of sequences from subtypes J, C and K are indicated

**Fig. 11:**
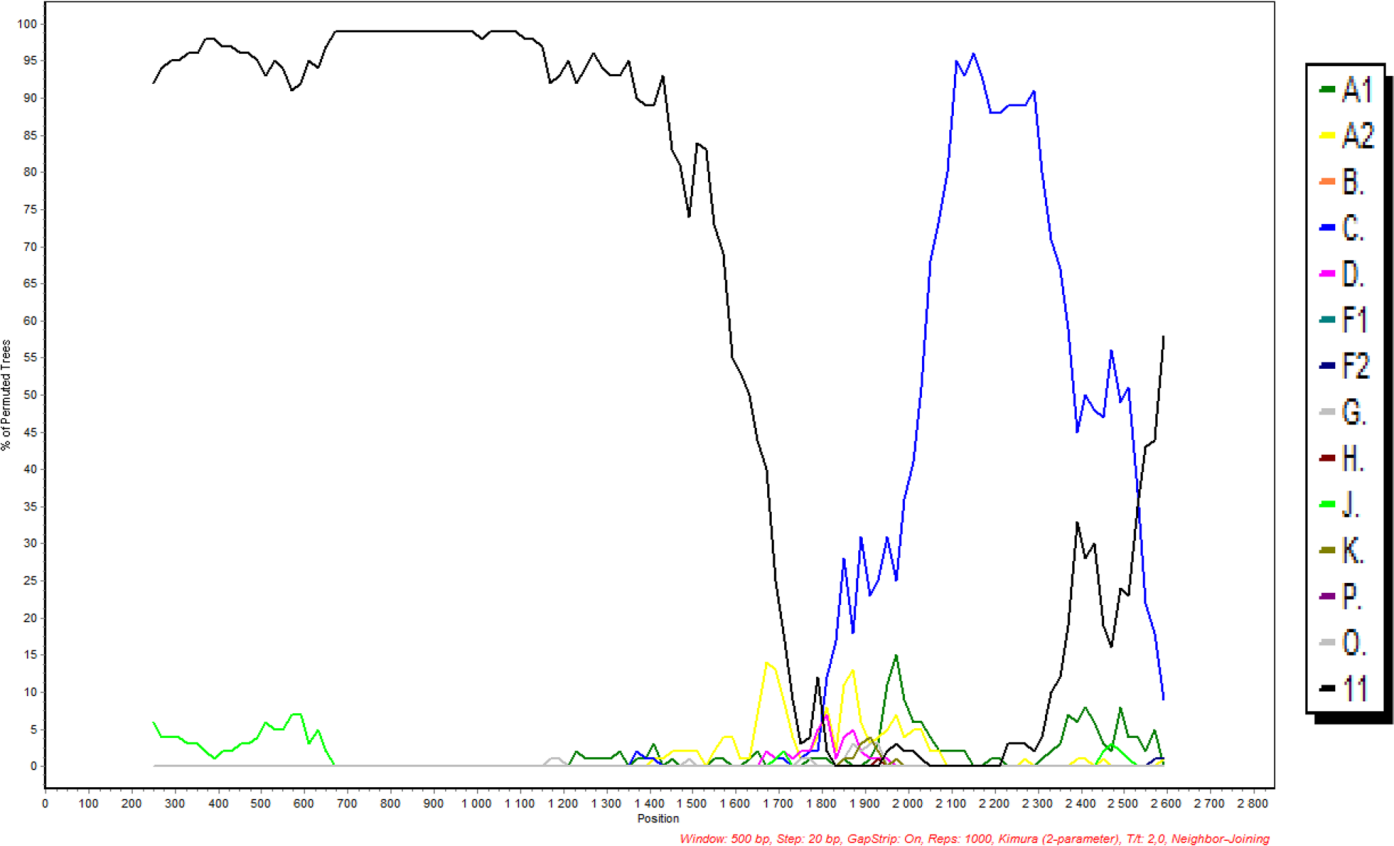
Simplot Boot scanning analysis of the pol (PR + RT + IN) sequence of 08MB26ZA. Scan confidence of pol gene analysis. Boot scan plot was generated with strict consensus on a window size of 500 bp, step size of 20 bp with gaps stripped. It was modelled with Kimura-2 parameter for 1000 replicates. The analysis confirmed the presence of CRF11_cpx and subtype C sequences

The gene fragments were also subjected to jumping Profile Hidden Markov Model (jPHMM) and Simplot analyses. On jPHMM analysis, the *gag*, e*nv* and *nef* sequences were assigned as related to subtype C, PR as related to subtype J, RT as J/C; and IN as C/A1. The *pol* (PR + RT + IN) sequence was determined as recombinant comprising sequences from subtypes J, C and K (Fig. 10). Further bootscan analysis by Simplot showed that the *pol* contained sequences from CRF11_cpx and subtype C viruses (Fig. 11); in agreement with the outcomes of the REGA, and the phylogenetic analysis in which the *pol* clustered with CRF11_cpx sequences. A summary of the outcomes of the subtyping and recombination analyses of the different gene regions of 08MB26ZA is shown in Table 1.

**Table 1:** Summary of subtype assignments by phylogenetic, REGA and jPHMM HIV subtyping and recombination analyses of 08MB26ZA

Gene	Phylogenetic analysis	REGA	jPHMM
*gag*	HIV-1 subtype C	HIV subtype C	HIV-1 subtype C
PR	HIV-1 CRF11_cpx	HIV subtype J	HIV-1 subtype J
RT	HIV-1 CRF11_cpx	HIV-1 CRF11_cpx	HIV-1 subtype J/C
IN	Outlier to HIV-1 subtype C	HIV-1 subtype C	HIV-1 subtype C/A1
*pol*	HIV CRF11_cpx	CRF11_cpx/HIV-1 subtype C	HIV-1 subtype J/C/K
*nef*	HIV-1 subtype C	HIV-1 subtype C	HIV-1 subtype C
*env* (C2-V5)	Outlier to HIV-1 subtype C	HIV-1 subtype C	HIV-1 subtype C

There was agreement among the analytic tools in inferring subtypes and recombination patterns for some of the genes regions and not for others. For example, jPHMM indicated the presence of subtype A1 sequences in the IN gene in addition to subtype C sequences; and also the presence subtype K sequences in the *pol* gene. The jPHMM indicated the presence of subtype J sequences in the *pol* gene in contrast to CRF11_cpx indicated by phylogenetic analysis and REGA. This discrepancy can however be reconciled in that subtype J sequences constitute a significant component of CRF11_cpx viruses. Consequently, the indication by phylogenetic analysis, REGA and Simplot tools of the presence of CRF11_cpx sequences in the *pol* gene, in addition to the assignment of the *gag*, *env* and *nef* genes to subtype C overwhelmingly suggests that virus 08MB26ZA harboured HIV-1 subtype C and HIV-1 CRF11_cpx sequences.

The partial characterization of a CRF11_cpx/HIV-1 subtype C-like virus from a 47 year old South African female is reported. The current analysis shows that the virus differed markedly from pure HIV-1 subtype J and CRF11_cpx in its sub-genomic assignments. It is only in the PR and RT genes that it exhibited a related pattern of mosaicism as seen in CRF11_cpx. The entire genome of CRF11_cpx is partitioned into sub-genomic regions comprising subtypes A, J, U, G and CRF01_AE (Paraskevis et al. 2000), and in 08MB26ZA subtype G and J sequences were also identified in the *pol* gene by REGA analysis. The *gag* of 08MB26ZA is related to subtype C, whereas in CRF11_cpx, it is derived from subtype A. Similarly, the IN, *env* C2-V5 and *nef* genes of 08MB26ZA are related to subtype C, whereas in CRF11_cpx the IN and *env* C2-V5 genes are derived from subtype A and *nef* is derived from CRF01_AE. HIV-1 subtype J and CRF11_cpx are predominantly found in the Central African region (Koyalta et al. 2009; Torimiro et al. 2009; Ndembi et al. 2008; Caron et al. 2012), with few reports from Southern Africa (Angola and Zambia) on the presence of subtype J viruses (Trask et al. 2002; Yebra et al. 2013; Bartolo et al. 2009).

Availability of the complete genome would have afforded a detailed understanding of the mosaic nature of virus 08MB26ZA allowing for a full comparison with CRF sequences in the sequence database. However, a search of the Los Alamos HIV sequence database did not reveal any variant that contains HIV-1 subtype C and CRF11_cpx sequences in its genomic composition. In another vein, since population based sequencing was employed it is not possible to rule out dual infection with HIV-1 subtype C and CRF11_cpx. However, CRF11_cpx has not been reported from South Africa. It is also understood that the patient from whom the virus under investigation was recovered had never travelled out of South Africa, increasing the likelihood that the observation could be an infection with a recombinant CRF11_cpx/HIV-1-C like virus. It is important to note that this is a description of a virus from a single individual harboring genes from an HIV circulating recombinant form (CRF11_cpx) and a pure HIV subtype (HIV-1 subtype C). Therefore, the implications in terms of its epidemiologic and biologic preferences are unknown.

### Genotypic drug resistance and co-receptor usage analyses

Drug resistance genotypic analysis of the PR, RT and IN genes did not reveal the presence of primary or major drug resistance mutations. However, examination of the PR revealed the following amino acid substitutions; L10V, T12P, K14R, I15 V, G16A, A28P and T31R and V32L; while in the RT the following amino acid amino acid substitutions I5 V, E6D, V35T, T39L, K122 N, K173T, D177G, T200E, I202V, R211K, V245N, E248D, D250E, A272P, E293 V, I329 V, Q334L, G335D, R356K, M357R, R358K, G359A, K366R, T369A, A371V, T377L, K390R, K395R, A400T, E435A, and A437V were observed in comparison with the global subtype B consensus sequence. Similarly, the following amino acid substitutions; K14R, S24N, D25E, V31I, M50I, I72V, V99F, F100Y, T112V, T124N, T125A, K136Q, V201I, Y227F, L234I, N254K, S255G, and S283G were observed in the IN when compared with the global subtype B consensus sequence. These amino acid changes are of unknown phenotypic significance. However, in the absence of primary or major drug resistant associated mutations it is expected that virus 08MB26ZA would be susceptible to currently used antiretroviral (ARV) drugs in South Africa.

The V3 loop is a major determinant for viral tropism and co-receptor usage. Analysis of the V3 loop of 08MB26ZA showed it had the GPGQ tetrapeptide motif and co-receptor usage prediction with webPSSM showed it was a CCR5 virus with a net charge of +3. This co-receptor prediction suggests that the virus would be susceptible to entry inhibitors such as Maraviroc.

## Conclusion

The identification of a CRF11_cpx/HIV-1 subtype C-like recombinant virus from South Africa, a region with an overwhelming predominance of HIV-1 subtype C, has been described. This is a signal suggesting the likely introduction of new and complex genetic variants in South Africa; and highlights the importance for regular genetic diversity studies due to potential implications for diagnosis. Apparently, this is the first report of a putative CRF11_cpx/HIV-1 subtype C unique recombinant from South Africa.

## Methods

### Patient information

Blood was collected from a 47 year old female (08MB26ZA) from Capricorn district, South Africa in 2008. At the time of blood collection, the patient was not on ARV. The CD4+ cell count and plasma viral load measurements were not available. The route of infection was heterosexual and the probable year of infection was 2005.

### Research ethics considerations

The study protocol was approved by the Safety, Health and Research Ethics Committee of the University of Venda, South Africa. Signed informed consent was obtained before blood sample and demographic data were collected.

### Generation of gene regions by PCR

Viral DNA was obtained from patient’s peripheral blood mononuclear cells using QIAamp DNA kit (Qiagen, Valencia, CA) according to the manufacturer’s instructions. The *gag, pol*, *env* C2-V5 and *nef* genes were amplified by nested PCR. The first round PCR of the different fragments were amplified using one of the following primer pairs; GagDrev 5′-AAT TCC TCC TAT CAT TTT TGG -3′ and Gag Dforw 5′-TCT CTA GCA GTG GCG CCC G-3′ for *gag*; Pol1C; 5′-GAA GGA CAC CAA TTG AAA GAC TGC AC-3′ and INrev1; 5′-TCT CCT GTA TGC AGA CCC CAA TAT-3′ for *pol*, while ED5;5′-ATG GGA TCA AAG CCA TGT G-3′ and ED12; 5′-ATG GCT TCC TGC TCC CAA GAA CCC AAG-3′ were used for the *env* C2-V5; and SQ15FC; 5′-GAG AGC GGT GGA ACT TCT-3′ and NefOR; 5′-AGG CAA GCT TTA TTG AGG-3′ for the *nef* gene.

The amplifications were carried out in a reaction mixture containing 5 µl of 10× buffer, 200 µM of dNTPs, 0.5 µM of each primer, 0.5 U of Taq Expand Long Template, and 10 µl of DNA template in a final reaction volume of 50 µl. The cycling conditions used for all the reactions were as follows: 94 °C for 3 min followed by 35 cycles of 94 °C for 30 s, 55 °C for 45 s and 72 °C for 2 min; and a final extension for 10 min at 72 °C. The nested reaction was carried out in a reaction mixture and cycling conditions as outlined above but with the following primer pairs *Gag* Aforw 5′-CTC TCG ACG CAG GAC TCG GCT T-3′ and GagCrev; 5′-TCTTCTAATACTGTATCATCTGC-3′ for the *gag* gene; GagP1 5′-CAAGGG GAGGCCAGGGAATTT-3′ and INrevII 5′-CCTAGTGGGATGTGTACTTCTGA-3′ for the *pol* gene; ES7 5′-CTGTTAAATGGCAGTCTAGC-3′ and ES8 5′-CACTTCTCC AATTGTCCCTCA-3′ for *env* C2–V5; and Nefforw 5′-CCTAGAAGAATAAGACAG GGCTT-3′ and Nefrev 5′-CCTGGAACGCCCCAGTGG-3′ for the *nef* gene region. The amplified products were purified and directly sequenced using a BigDye Terminator sequencing kit (Applied Biosystems, Foster City, CA) making use of appropriate sequencing primers for the *gag* and *pol* genes while the *env* and *nef* were sequenced with their nested primers. The sequenced fragments from both strands were assembled and edited using SeqMan Pro and Seqbuilder program contained in the DNAStar software version 7 (DNASTAR, INC, Madison, Wisconsin, USA).

### Phylogenetic and recombination analyses

The *gag*, *pol*, PR, RT, IN, *env* C2-V5, and *nef* sequences were aligned with pure subtypes A–D, F–H, J, K, and CRF11_cpx reference sequences obtained from the Los Alamos database. Test and reference sequences were codon aligned using Muscle and phylogenetic trees generated by the maximum likelihood method as implemented in MEGA version 5.2 with 1000 bootstrap replicates to estimate the reliability of the branching clusters (Tamura et al. 2011). The *gag* and *pol* sequences were additionally analyzed for recombination and CRF with REGA HIV Subtyping tool version 3.0 (Peña et al. 2012). The REGA tool employs phylogenetic and bootscanning methods to assess the query sequence against a mirror of pure subtypes and CRFs. Additionally, the *pol* sequence was analysed by jPHMM and Simplot to confirm recombination.

### Determination of genotypic drug resistance and co-receptor usage

The PR, RT and IN nucleotide sequences were also submitted to the Stanford HIV Drug Resistance Interpretation Algorithm for the detection of mutations associated with drug resistance and closest subtype identity (Stanford HIV drug resistance mutation database). The V3 loop of the *env* gene is a major determinant for viral tropism and co-receptor usage. The predicted amino acid sequences of the V3 loop of virus 08MB26ZA was submitted to the webPSSM, an online interactive program that predicts co-rector usage of viruses by calculating the net charge of the V3 loop and makes predictions on probable co-receptor usage (WebPSSM).

### GenBank accession numbers

The gene regions described here have been deposited in GenBank under the following accession numbers: HM049909 (*gag*); GU188817 (PR); HM049908 (RT); HM049906 (IN); HM049910 (*env* C2-V5); HM049907 (*nef)*.
